# Unlocking the Potential of *Curcumae Rhizoma* Aqueous Extract in Stress Resistance and Extending Lifespan in *Caenorhabditis elegans*

**DOI:** 10.3390/molecules30081668

**Published:** 2025-04-08

**Authors:** Linyao Jing, Yanlin Zhao, Lijun Jiang, Fei Song, Lu An, Edmund Qi, Xueqi Fu, Jing Chen, Junfeng Ma

**Affiliations:** 1 State Key Laboratory for Diagnosis and Treatment of Severe Zoonotic Infectious Diseases, Key Laboratory for Zoonosis Research of the Ministry of Education, School of Life Sciences, Jilin University, Changchun 130012, China; jinglinyao163@163.com (L.J.); 13194257525@163.com (Y.Z.); 18844111915@163.com (F.S.); anlu22@mails.jlu.edu.cn (L.A.); 18943170033@163.com (E.Q.); fxq@jlu.edu.cn (X.F.); 2 Changchun Heber Biological Technology Co., Ltd., Changchun 130012, China; jianglijun850419@163.com

**Keywords:** network pharmacology, stress resistance, *Caenorhabditis elegans*, IIS, ROS, molecular docking

## Abstract

The enhancement of stress resistance is crucial for delaying aging and extending a healthy lifespan. Traditional Chinese medicine (TCM), a cherished treasure of Chinese heritage, has shown potential in mitigating stress and promoting longevity. This study integrates network pharmacology and in vivo analysis to investigate the mechanisms and effects of *Curcumae Rhizoma* (*C. Rhizoma*), known as “E Zhu” in Chinese. Ultra-Performance Liquid Chromatography–Tandem Mass Spectrometry (UPLC-MS/MS) identified 10 active compounds in its aqueous extract, interacting with 128 stress-related targets. Gene Ontology (GO) and Kyoto Encyclopedia of Genes and Genomes (KEGG) analyses revealed pathways such as stress response, FoxO signaling, and insulin resistance. In *Caenorhabditis elegans*, 10 mg/mL of *C. Rhizoma* aqueous extract improved resistance to UV, thermal, oxidative, and pathogen-induced stress, extending lifespan in a dose-dependent manner. Mechanistically, it reduced reactive oxygen species (ROS), increased superoxide dismutase (SOD) activity, and enhanced UV resistance via the insulin/IGF-1 pathway and DAF-16 translocation. Molecular docking highlighted hexahydrocurcumin (HHC) and related compounds as key bioactives. Furthermore, we also observed that *C. Rhizoma* aqueous extract significantly extended both the lifespan and healthspan of nematodes. These findings highlight the potential of *C. Rhizoma* in stress mitigation and longevity promotion, offering valuable insights into the therapeutic applications of TCM.

## 1. Introduction

Chronic exposure to adverse conditions accelerates biological aging and heightens the risk of age-related diseases [1]. Persistent UV radiation, a physical stressor, depletes skin stem cells and leads to premature skin aging [2,3,4]. In extreme cases, it may even escalate to skin cancer [5,6]. UV radiation can trigger an overproduction of ROS within the body [7,8], which further damage DNA by disrupting its structural integrity [9]. Additionally, excess ROS induces lipid peroxidation and the oxidation of amino acid side chains, exacerbating the risk of age-related conditions like Alzheimer’s disease [10,11,12].

Recent research has highlighted the stress-alleviating effects of traditional Chinese medicine (TCM), establishing them as a cutting-edge focus in the field [13]. Herbs such as *Panax ginseng*, *Aloe vera*, *Rhodiola rosea*, *Scutellaria baicalensis*, *Glycyrrhiza uralensis* and *Schisandra chinensis* are noted for their anti-stress benefits [14,15,16,17,18]. Bioactive constituents in TCM, including polysaccharides, polyphenols, flavonoids, saponins, terpenes, and aromatic compounds, are recognized for their protective effects against stress [19,20]. *Curcumae Rhizoma* (*C. Rhizoma*), known as “E Zhu” in Chinese, is derived from the dried rhizomes of *Curcuma phaeocaulis* Val., *Curcuma kwangsiensis* S.G. Lee et C.F. Liang, or *Curcuma wenyujin* Y.H. Chen et C. Ling [21], and is officially listed in the 2020 Edition of the *Chinese Pharmacopoeia* (ChP). Studies have demonstrated that *C. Rhizoma* possesses anti-inflammatory and antioxidant properties, and it has been reported to alleviate oxidative stress in the liver of golden pompano [22]. Recent research has identified the principal bioactive components of *C. Rhizoma*, including aromatic volatile oils and curcuminoid compounds [23]. Specifically, the curcuminoid compounds, i.e., curcumin, demethoxycurcumin, and bisdemethoxycurcumin, constitute 75%, 18%, and 7%, respectively, and are renowned for their antioxidant, anticancer, and anti-inflammatory properties [24,25,26].

With the advent of systems biology, network pharmacology has become a crucial tool for exploring drug mechanisms since its inception in 2007. This approach analyzes the intricate networks connecting drugs, diseases, and biomolecules, offering a comprehensive view of drug effects [27,28]. Despite its potential, network pharmacology faces challenges, including difficulties in identifying new compounds and targets, and a lack of advanced methods for assessing dosage and synergistic effects of traditional Chinese medicine compounds. Addressing these issues requires suitable animal models for the in vivo validation of network pharmacology predictions. *Caenorhabditis elegans* (*C. elegans*), a classic model organism, is highly valued for its short life cycle, genetic tractability, and conserved biological mechanisms with humans. It excels in disease modeling, drug screening, and generating vital data for drug toxicity and efficacy evaluations [29].

**Figure 1:**
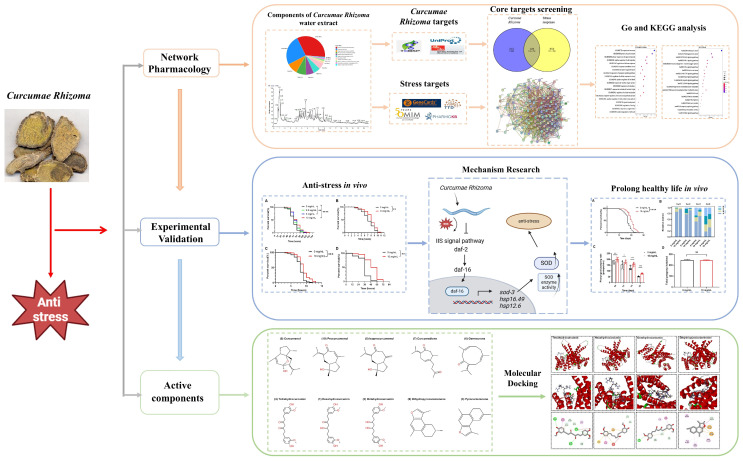
Content summary diagram, including network pharmacology target prediction, in vivo experiment verification, effectiveness of active components, and molecular docking target prediction. Statistical significance is indicated as * *p* < 0.05, ** *p* < 0.01, *** *p* < 0.001, **** *p* < 0.0001.

This research aims to investigate the protective effects of *C. Rhizoma* against stress. Utilizing network pharmacology, we firstly identify potential molecular targets and pathways. Then, these findings are validated in vivo by employing *C. elegans* as a biological model, as illustrated in Figure 1. This interdisciplinary approach combines network pharmacology with nematode experimentation, bridging molecular intricacies to whole-organism responses and thereby pioneering a novel method to understand drug mechanisms. This synergistic methodology establishes an advanced platform for disease modeling, streamlining drug discovery and refinement processes. It significantly advances the field of TCM, elucidating the mechanisms underlying the efficacy of herbal compounds like *C. Rhizoma*. This research paves the way for a deeper understanding of TCM’s therapeutic capabilities.

## 2. Results and Discussion

### 2.1. Network Pharmacology Reveals the Anti-Stress Potential of C. Rhizoma

**Figure 2:**
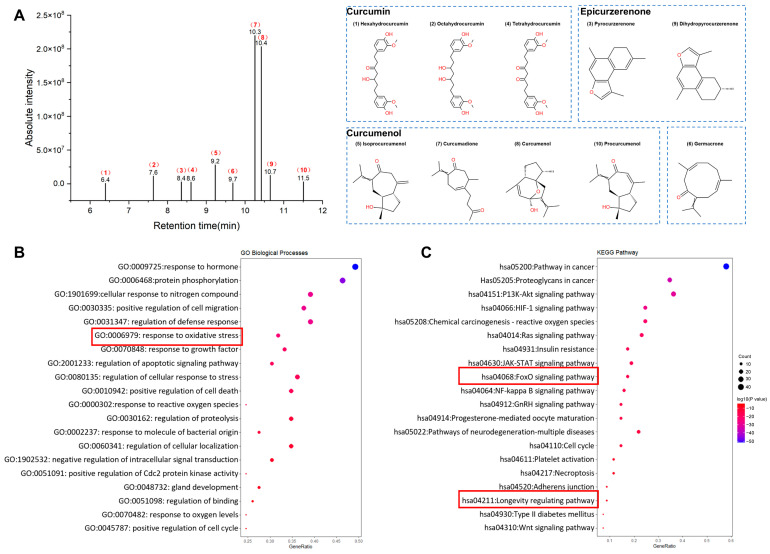
(**A**) The retention time and absolute intensity of the target compound in the water extract of *C. Rhizoma.* The red numbers on the left image correspond one-to-one with the numbers in front of the compounds in the right image. (**B**) GO enrichment analysis of potential targets in *C. Rhizoma* for biological processes, the red rectangle represents the biological processes related to the response to oxidative stress involved in the intersection targets between *C. Rhizoma* and stress. (**C**) KEGG analysis, the FOXO signaling pathway and the longevity signaling pathway highlighted by the red rectangles are the key pathways of our focus.

We identified the chemical constituents of *C. Rhizoma* using the Traditional Chinese Medicine Systems Pharmacology (TCMSP) platform with parameters of oral bioavailability (OB) ≥ 30% and drug-likeness (DL) ≥ 0.18, resulting in ingredients that met the criteria (Supplementary Table S1). At the same time, pharmacokinetic parameter screening was conducted using the Swiss ADME platform, and 15 compounds met the criteria (Supplementary Table S2). We conducted a metabolomic analysis of an aqueous extract of *C. Rhizoma* using UPLC-MS/MS. A total of 766 metabolites were identified, and classification based on the Classfire database revealed that 33.59% were lipids, 9.28% were organoheterocyclic compounds, and 8.24% were phenylpropanoids and polyketides, along with other categories such as organic acid derivatives and alkaloid derivatives (Supplementary Figure S1). The original total ion chromatogram is shown in Supplementary Figures S2 and S3. Integrating the active ingredient screening results from the TCMSP and Swiss ADME databases, four categories were identified as important active ingredients in the aqueous extract of *C. Rhizoma*, in Table 1. Curcumenol-type compounds, including curcumenol, procurcumenol, isoprocurcumenol, and curcumadione, were identified. Another category comprised curcumin-related compounds, such as tetrahydrocurcumin (THC), hexahydrocurcumin (HHC), and octahydrocurcumin (OHC), which are hydrogenated derivatives of curcumin. Additionally, epicurzerenone-type compounds, including dihydropyrocurzerenone and pyrocurzerenone, were detected, along with germacrone, which belonged to a separate category. These four categories of compounds were confirmed in ion chromatograms based on their retention times, with the results shown in Figure 2A.

Using Swiss Target Prediction, we predicted targets for these components, yielding 215 unique targets. An exhaustive search in OMIM, GeneCards, TTD, and PharmGKB databases identified 854 genes associated with stress. Through a Venn diagram, we identified 128 intersecting genes with the *C. Rhizoma* dataset (Supplementary Figure S4). The STRING database was utilized to construct a PPI network, using median cutoffs for degree, betweenness centrality, and closeness centrality to identify core targets. The analysis revealed numerous key targets, with pivotal nodes including AKT1, TNF, EGFR, SRC, HSP90AA1, MAPK3, STAT3, and MTOR (Supplementary Figure S5 and Table 2). Among these, AKT1 exhibits the highest network centrality (degree: 91) and plays a crucial role in stress adaptation via the PI3K-Akt pathway—regulating cell survival, inhibiting apoptosis, reprogramming metabolism, defending against oxidative stress, and promoting autophagy [30]. Similarly, two other top-ranked targets, MAPK3 and MTOR, also play crucial roles in stress responses. MAPK3 facilitates cell survival by activating transcription factors that drive proliferation and repair mechanisms while modulating antioxidant defenses, whereas MTOR integrates signals related to energy, nutrients, and oxidative stress to orchestrate cellular adaptation [31,32].

GO and KEGG enrichment analyses using Metascape (*p* < 0.01) highlighted the roles of core targets in stress responses, including pathways for nitrogen response, oxidative stress, HIF-1 signaling, insulin resistance, FoxO signaling, neurodegeneration, and longevity (Figure 2B,C). These findings underscore the potential of *C. Rhizoma* in anti-stress responses.

Network pharmacology is essential for identifying the active ingredients in traditional Chinese medicine and elucidating their mechanisms of action, thereby clarifying their therapeutic effects and interactions with diseases [33]. Combining network pharmacology with UPLC-MS/MS validation is an effective approach to further explore and verify the active components of a compound. This method enhances the accuracy and reliability of the results, providing a more comprehensive understanding [34]. According GO and KEGG enrichment analyses, the results highlighted the roles these targets play in key stress-responsive pathways, including the Hypoxia-Inducible Factor 1 (HIF-1) pathway, a key regulator of cellular hypoxic responses [35,36], and the FoxO signaling pathway, which influences stress resistance and promotes longevity [37,38,39]. These findings underscore the broad-spectrum anti-stress potential of *C. Rhizoma*, suggesting its capacity to modulate diverse biological processes in response to stress.

### 2.2. Aqueous Extract of C. Rhizoma Boosts C. elegans Stress Resilience

**Figure 3:**
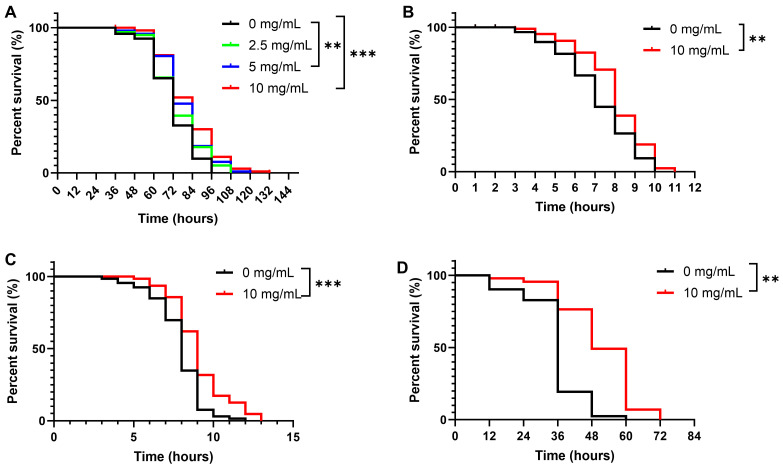
Effects of Curcuma Rhizome on the wild-type *C. elegans* (N2 strain) under various stress conditions: (**A**) UV stress, (**B**) heat stress, (**C**) oxidative stress, (**D**) infection stress by *Pseudomonas aeruginosa.* Statistical significance is indicated as ** *p* < 0.01, *** *p* < 0.001.

To assess the impact of *C. Rhizoma* aqueous extract on *C. elegans’* stress resistance, nematodes were exposed to various stresses, including UV radiation, thermal stress, oxidative stress, and *Pseudomonas aeruginosa* infection. Nematodes grown on NGM agar plates with extract concentrations of 0, 2.5, 5, and 10 mg/mL exhibited a dose-dependent increase in UV stress resistance, with lifespan extensions of 4.06%, 8.76%, and 12.99%, respectively (Figure 3A). Meanwhile, under thermal stress at 35 °C, nematodes treated with 10 mg/mL *C. Rhizoma* aqueous extract exhibited an 11.57% increase in average lifespan, indicating enhanced thermotolerance (Figure 3B). Similarly, the same concentration increased the average lifespan by 19.82% under oxidative stress (Figure 3C). Furthermore, *C. Rhizoma* significantly improved survival against *Pseudomonas aeruginosa* infection in nematodes (Figure 3D).

*C. Rhizoma* has demonstrated various pharmacological properties in vitro, including antibacterial, antioxidant, and anti-inflammatory activities [40,41]. It has been shown to mitigate liver oxidative stress in golden pompano [42,43]. Its active component germacrone has the effect of alleviating cardiac remodeling by modulating PI3K/AKT-mediated oxidative stress, inflammation and apoptosis [44]. Our in vivo studies confirmed the protective role of *C. Rhizoma*, evidenced by enhanced stress resistance in nematodes exposed to UV radiation, thermal stress, oxidative stress, and *Pseudomonas aeruginosa* infection. These findings are significant given the established link between stress and various diseases and the growing interest in natural products with antioxidant properties.

### 2.3. C. Rhizoma Aqueous Extract Diminished ROS Levels and Elevated SOD Activity Under UV Stress

**Figure 4:**
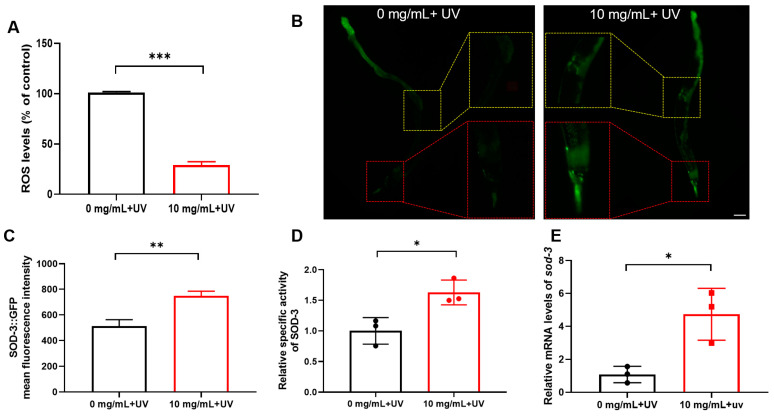
The effects of *Curcuma Rhizome* aqueous extract on oxidative stress indicators in *C. elegans* under UV stress: (**A**) Levels of ROS in N2 nematodes after UV stress, expressed as a percentage of untreated control (set as 100%). (**B**) The expression of SOD-3 in CF1553 nematodes. The red box represents a magnified view of the fluorescence intensity in the head region of CF1553 mutant nematodes, while the yellow section indicates a magnified view of the vulva region. The scale bar represents 100 μm. (**C**) The average fluorescence intensity of CF1553 by Image J. (**D**) SOD-3 enzyme activity in N2 nematodes following UV stress. (**E**) Expression levels of the *sod-3* gene in N2 nematodes under UV stress. Statistical significance is indicated as * *p* < 0.05, ** *p* < 0.01, *** *p* < 0.001.

UV rays, among the most harmful elements of solar radiation, can intensify cellular damage and incite inflammation [45,46,47,48,49]. In addition, UV radiation is a convenient method for inducing stress, and 10 mg/mL *C. Rhizoma* aqueous extract has shown efficacy in resisting various stresses. Therefore, this combination was employed to elucidate the mechanism by which *C. Rhizoma* enhances stress resistance in nematodes. As elevated levels of ROS are a pivotal contributor to stress within biological systems [50], the ROS level was firstly assessed. Our experimental findings revealed that *C. Rhizoma* aqueous extract significantly reduced ROS levels in nematodes under UV stress. Specifically, ROS content decreased by 71% compared to the control group, highlighting the potent antioxidant properties of the *C. Rhizoma* extract (Figure 4A).

Throughout evolution, nematodes have developed an antioxidant defense system that includes the superoxide dismutase family. The SOD-3 enzyme converts superoxide radicals into hydrogen peroxide, which is further broken down into water by other antioxidant enzymes, maintaining a dynamic balance of free radicals. The nematode strain CF1553, with GFP-marked *sod-3*, shows fluorescence in the head, tail, and vulva. The magnified images clearly demonstrate that the treated group exhibits enhanced fluorescence in the head and vulva regions compared to the untreated group. Image J software (V1.8.0.345) analysis further confirmed that the *C. Rhizoma* aqueous extract significantly increased SOD-3 expression. Specifically, the average fluorescence intensity rose from approximately 500 in the untreated group to 700 in the treated group, representing an increase of about 40% (Figure 4B,C). Enzyme activity assays showed that SOD activity was 1.62 times higher in the *C. Rhizoma*-treated nematodes (Figure 4D). Furthermore, qPCR indicated that *sod-3* expression was elevated 3.67 times in the treated group compared to the control (Figure 4E).

ROS are key catalysts of skin aging due to UV stress, with 1.5% to 5.0% of skin-utilized oxygen converting into ROS [51,52,53]. Excessive superoxide anion production impairs various cellular functions and accelerates aging processes triggered by radiation [54]. To counteract ROS damage, the body activates an antioxidant defense mechanism involving enzymes like superoxide dismutase (SOD), glutathione peroxidase (GSH-Px), and catalase (CAT) [55]. *C. elegans* has five *sod* genes, with *sod-3* encoding mitochondrial Mn-SOD regulated by *daf-2* and *daf-16* genes [56]. As shown in Figure 4, the pretreated *C. Rhizoma* aqueous extract significantly reduced UV-induced ROS levels. It also upregulated *sod-3* gene expression and protein levels, enhancing SOD activity, thereby elucidating *C. Rhizoma*’s mechanism in combating UV stress.

### 2.4. C. Rhizoma Potentiated Nematode UV Resistance via Insulin/IGF-1 Signaling Pathway

**Figure 5:**
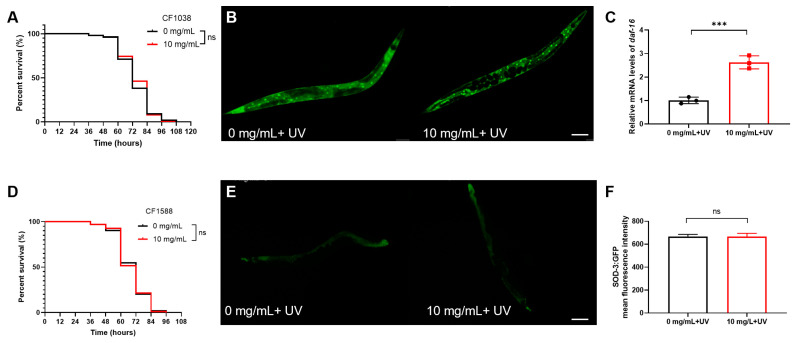
Enhancement of UV stress resistance in *Caenorhabditis elegans* by *Curcuma Rhizome* through the insulin-like signaling pathway. (**A**) Lifespan under UV stress in CF1038 (*daf-16* mutant strain). (**B**) Nuclear translocation of DAF-16 in TJ356 nematodes under UV stress, Scale bar, 100 μm. (**C**) Expression levels of the *daf-16* gene in N2 nematodes under UV stress. (**D**) Lifespan under UV stress in CF1588 (*daf-16/daf-2* double-mutant strain). (**E**) Impact of *Curcuma Rhizome* aqueous extract on expression of SOD-3 in CF1588 nematodes after UV radiation, Scale bar, 100 μm. (**F**) Image J analysis of mean fluorescence intensity in CF1588 nematodes. Statistical significance is indicated as *** *p* < 0.001.

Research indicates that the insulin/IGF-1 signaling (IIS) pathway is pivotal in regulating nematode longevity and stress resistance [52,53]. Within this pathway, DAF-2 (an insulin-like receptor) and DAF-16 (the ortholog of human FoxO in *C. elegans*) play crucial roles, with DAF-16 acting downstream of DAF-2 [54]. To evaluate the mechanism by which *C. Rhizoma* enhances nematode UV resistance, the *daf-16* mutant strain CF1308 was firstly employed. The results indicated that *C. Rhizoma* did not extend the lifespan of UV-exposed CF1308 nematodes (Figure 5A). Subsequently, the effect of *C. Rhizoma* on DAF-16 localization was investigated using the transgenic nematode strain TJ356, which expresses the DAF-16::GFP protein. Under normal conditions, DAF-16::GFP is primarily distributed in the cytoplasm, exhibiting a uniform diffuse fluorescence signal. Under stress conditions, DAF-16::GFP naturally translocates to the nucleus to some extent, resulting in distinct nuclear fluorescence aggregation. However, in the *C. Rhizoma* treatment group, these aggregates appeared clearer and significantly increased in number. This suggests that the aqueous extract of *C. Rhizoma* can more effectively promote the translocation of DAF-16 from the cytoplasm to the nucleus, thereby enhancing the activation of downstream antioxidant enzymes and providing better protection against UV-induced stress. Notably, *C. Rhizoma* significantly promoted the translocation of DAF-16 from the cytoplasm to the nucleus (Figure 5B and Supplementary Figure S6). Upon exposure to identical UV stress conditions, the expression level of the *daf-16* gene was elevated by 2.6-fold in the *C. Rhizoma*-treated group compared to the control group (Figure 5C).

Consistent with the findings in the *daf-16* mutant, *C. Rhizoma* also failed to extend the lifespan of the *daf-2* and *daf-16* double-mutant nematode CF1588 under UV stress (Figure 5D). CF1588 also expresses the SOD-3::GFP protein, with *sod-3* being a downstream target of *daf-16* and encoding a superoxide dismutase enzyme that mitigates stress-induced damage. In the double-mutant strain, there was no significant difference in SOD-3 expression levels between the *C. Rhizoma*-treated and control groups (Figure 5E,F). These findings collectively suggest that *C. Rhizoma* enhances UV stress resistance in nematodes through a mechanism dependent on the *daf-2* and *daf-16* genes, potentially involving the insulin signaling pathway.

The intricate signaling pathways that combat the aging process and mitigate oxidative stress are multifaceted, notably involving the IIS pathway. This pathway is crucial for regulating cellular metabolism and longevity [55] and is a central mechanism for anti-aging and anti-stress in the nematodes [38]. The IIS pathway begins at the DAF-2 insulin receptor, continues through AGE-1/PI3K, and activates the AKT-1/2 kinases, ultimately leading to the activation of the DAF-16/FoxO transcription factor [56]. This cascade is pivotal for modulating the lifespan and metabolic homeostasis of *C. elegans*. Studies consistently show that mutants with disruptions in the *daf-2* gene exhibit an extended lifespan and enhanced oxidative stress resistance [57]. Conversely, *daf-16* mutants tend to have a shortened lifespan, highlighting the negative regulation of this transcription factor by insulin-like signaling.

As a key transcriptional regulator, DAF-16 is crucial for modulating the lifespan of *C. elegans*. Its vertebrate counterpart, FoxO, shares similar roles in longevity regulation [58]. Normally, DAF-16 is found in the cytoplasm, but under stress, it undergoes phosphorylation and activation, followed by translocation to the nucleus. This nuclear accumulation allows DAF-16 to regulate downstream target genes [59], including those encoding antioxidant enzymes like SOD-3 and heat shock proteins such as *hsp-16.49* and *hsp-12.6*, which are vital for stress response and survival [60].

### 2.5. Molecular Docking Analysis of Curcumin Derivatives as Key Active Compounds for Anti-UV Stress Effects Through FoxO Binding

**Figure 6:**
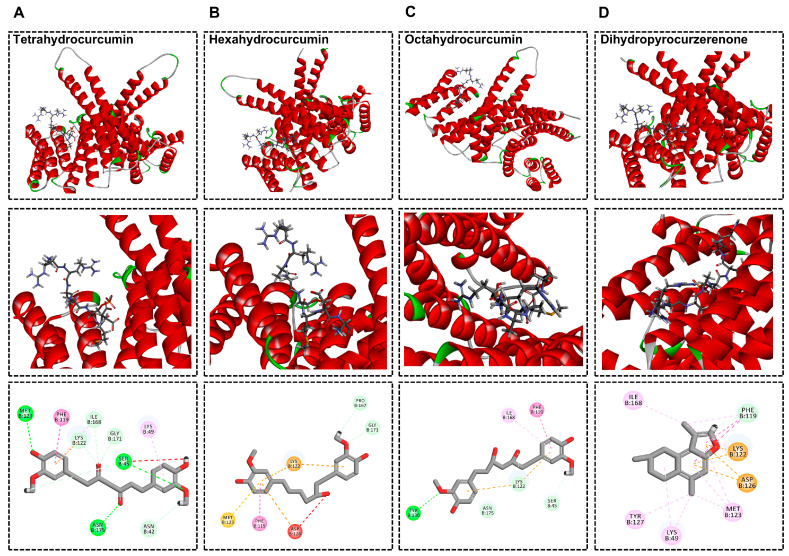
The molecular docking of tetrahydrocurcumin (**A**), hexahydrocurcumin (**B**), octahydrocurcumin (**C**), and dihydropyrocurzerenone (**D**), with the target protein FoxO, accompanied by their respective 3D and 2D schematic representations.

To further investigate the key active compounds responsible for the anti-UV stress effects of *C. Rhizoma*, molecular docking analyses were performed to evaluate their interactions with FoxO, the human homolog of *C. elegans* DAF-16. FoxO plays a crucial role in regulating stress resistance and longevity [61]. The results revealed that curcumin derivatives, specifically THC, HHC and OHC, showed significantly stronger binding affinities to FoxO, with binding energies of −217.427 kcal/mol, −200.956 kcal/mol, and −181.765 kcal/mol, respectively. In contrast, curcumenol, procurcumenol, isoprocurcumenol, curcumadione and other compounds (dihydropyrocurzerenone, pyrocurzerenone, and germacrone) showed weaker binding energies, suggesting less stable interactions with FoxO (Supplementary Figure S7). In molecular docking, a larger absolute value of binding energy indicates a more stable ligand–receptor complex and stronger binding [62]. The higher binding affinities of curcumin derivatives were attributed to diverse non-covalent interactions, including carbon–hydrogen bonds, Pi–cation interactions, Pi–anion interactions, Pi-Pi stacking, Pi–alkyl bonds, and Pi–sulfur bonds (Figure 6A–D).

Curcumin derivatives, THC, HHC, and OHC, are notable metabolites formed through curcumin reduction. THC, as a primary metabolite [63], demonstrates stronger antioxidant activity than curcumin, enhanced 2,2-diphenyl-1-picrylhydrazyl (DPPH) scavenging ability, and the capacity to upregulate glutathione peroxidase, glutathione S-transferase, and nicotinamide adenine dinucleotide phosphate (NADPH) quinone reductase [64]. In addition, THC exhibits improved water solubility and bioavailability compared to curcumin [65], although its absorption and distribution remain limited. HHC, formed by a further reduction in THC, shows even greater antioxidant activity by scavenging free radicals and protecting cells from oxidative damage [66]. OHC, the final reduced derivative of curcumin, surpasses curcumin in anti-tumor and anti-inflammatory effects [67], although its poor bioavailability has limited its study. Previous studies have also revealed that curcumin can prevent UV-induced photodamage at the cellular level [68], suppress oxidative stress caused by UV exposure, and inhibit apoptosis in A431 cells [69]. However, the anti-UV stress effects of curcumin derivatives such as tetrahydrocurcumin, hexahydrocurcumin, and octahydrocurcumin have not yet been investigated. Future research should focus on exploring the anti-stress potential of these three derivatives, evaluating their effects in combination, and comparing their efficacy to the overall therapeutic benefits of *C. Rhizoma*. This could provide valuable insights into their roles as potential therapeutic. Additionally, further studies could investigate the interactions between curcumin derivatives and a broader range of effector proteins within the insulin signaling pathway, including AKT1, to fully elucidate the mechanisms underlying their observed anti-UV stress effects.

### 2.6. C. Rhizoma Aqueous Extract Extended C. elegans Lifespan and Improved Health Indicators

**Figure 7:**
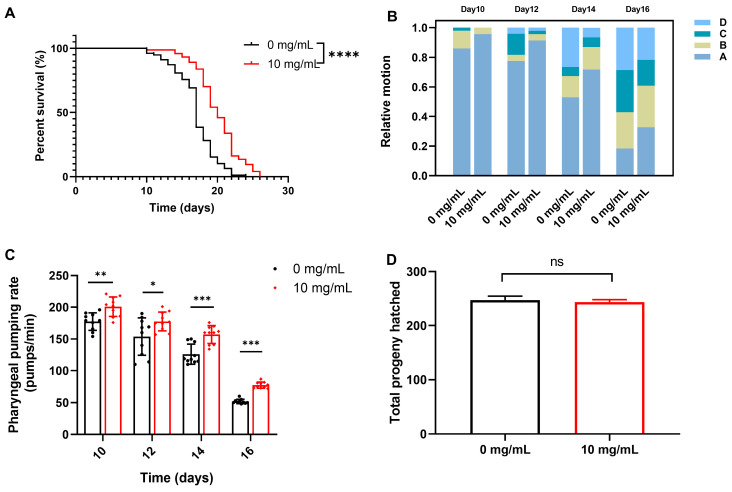
Effects of *C. Rhizoma* aqueous extract on health indicators in *C. elegans* (**A**) lifespan, (**B**) locomotive ability, (**C**) pharyngeal pumping frequency, (**D**) reproductive capacity. Statistical significance is indicated as * *p* < 0.05, ** *p* < 0.01, *** *p* < 0.001, **** *p* < 0.0001.

Stress resistance is closely associated with anti-aging effects. Studies have shown that improving stress resistance can not only delay the aging process but also extend healthy lifespan, forming a fundamental biological basis for anti-aging [70]. To assess the effects of *C. Rhizoma* on nematode lifespan and health indicators, we measured survival time, motility, pharyngeal pumping frequency, and reproductive capacity. Compared to the control group, *C. Rhizoma* significantly extended the lifespan of N2 nematodes by an average of 18.153% (Figure 7A). Motility, an indicator of nematode health, typically declines with aging. *C. Rhizoma* aqueous extract at a concentration of 10 mg/mL enhanced nematodes motility, with the effect becoming more pronounced as the nematodes aged, indicating a delay in the decline in motility and an improvement in health status (Figure 7B). Additionally, the extract delayed the decline in pharyngeal pumping frequency compared to the control group (Figure 7C). There was no significant difference in the number of offspring between the *C. Rhizoma*-treated group and the control group, suggesting that the *C. Rhizoma* aqueous extract does not affect reproductive capacity (Figure 7D).

*C. elegans* offers numerous advantages as a model organism for studying aging that are difficult to match by other species. The lifespan of wild-type N2 worms, when cultured at 20 °C, is typically around 2–3 weeks. Worms older than 10 days exhibit significant signs of aging, including a decline in locomotion, reduced activity, the decreased frequency of body bends, and even a loss of spontaneous movement. Their reproductive capacity declines significantly after 5–7 days of age. Due to these physiological characteristics, *C. elegans* serves as an in vivo model for identifying natural antioxidants with anti-aging properties [70]. The observed enhancement of nematode lifespan and health indicators by *C. Rhizoma* aqueous extract (Figure 7) underscores its potential as a natural health product. The improvement in motility and pharyngeal pumping frequency, coupled with no adverse effect on reproductive capacity, suggests that *C. Rhizoma* can enhance the overall health and well-being of the organism.

## 3. Materials and Methods

### 3.1. Chemicals and Reagents

*C. Rhizoma* was sourced from Tong Ren Tang (Beijing, China). Juglone was obtained from Yuan Ye Biotechnology (Shanghai, China). TRIZOL, DNA Marker, TransScript One-Step gDNA Removal, cDNA Synthesis SuperMix and TransStart Top Green qPCR Super Mix were all supplied by TransGen Biotech (Beijing, China). The ROS assay kit was procured from Beyotime (Shanghai, China). Levamisole hydrochloride was purchased from Yuan Ye Biotechnology (Shanghai, China), and the SOD assay kit was acquired from Solarbio Science & Technology (Beijing, China).

### 3.2. Preparation of C. Rhizoma Solution

First, we weigh out 1 g of *C. Rhizoma* into a conical flask, add 40 mL of ultrapure water to soak for 30 min, then boil for 20 min. We filter the liquid through a cheesecloth into a new container. The step of boiling and filtration is repeated three times in total. The resulting aqueous extract is concentrated to a final volume of 10 mL, resulting a concentration of 100 mg/mL based on the initial dry weight.

### 3.3. Ultra-Performance Liquid Chromatography–Tandem Mass Spectrometry (UPLC-MS/MS) Analysis of C. Rhizoma Solution

The water extract of *C. Rhizoma* was added to a centrifuge tube, followed by an equal volume of methanol/acetonitrile (1:1, *v*/*v*). Ultrasound-assisted extraction, known for enhancing the extraction efficiency of bioactive compounds from plant materials, was employed as described in previous studies [71]. After homogenizing for 60 s, the mixture was sonicated for 30 min at a low temperature. It was centrifuged at 12,000 rpm for 10 min at 4 °C, then left at −20 °C for 1 h to precipitate proteins. After another centrifugation at 12,000 rpm for 10 min at 4 °C, the supernatant was vacuum-dried, re-dissolved in 0.1 mL of 50% acetonitrile, homogenized, and centrifuged again at 12,000 rpm for 10 min at 4 °C. The supernatant was collected for analysis [72].

The extracted sample was analyzed using a UPLC-Orbitrap-MS system (UPLC, Vanquish; Thermo Fisher Scientific, Waltham, MA, USA; MS, Q Exactive HF-X, Thermo Fisher Scientific, Waltham, MA, USA). The column used was Waters HSS T3 (100 × 2.1 mm, 1.8 μm) at 40 °C, with a flow rate of 0.3 mL/min and an injection volume of 2 μL. The solvent system included Milli-Q water (0.1% formic acid) and acetonitrile (0.1% formic acid) with a gradient as follows: 0 min, 100% phase A; 1 min, 100% phase A; 12 min, 5% phase A/95% phase B; 13 min, 5% phase A/95% phase B; 13.1 min, 100% phase A; and 17 min, 100% phase A. HRMS data were acquired on a Q Exactive HFX mass spectrometer(Thermo Fisher Scientific, Waltham, MA, USA) with ESI using Full-MS-ddMS2(Thermo Fisher Scientific, Waltham, MA, USA). The ESI parameters were as follows: spray voltage +3000 V/−2800 V, source temperature 350 °C, and ion transport tube temperature 320 °C. The scan range was 70–1050 Da with resolutions of 70,000 (primary) and 17,500 (secondary).

The raw data were first preprocessed using Progenesis QI software (V2.0, Waters Corporation, Milford, CT, USA) for baseline filtering, peak detection, peak matching, retention time correction, and peak alignment, resulting in a data matrix containing retention time peak intensity and mass-to-charge ratio (m/z). In mass spectrometry, mass accuracy refers to the difference between the measured m/z value of an ion and its theoretical m/z value, typically expressed in parts per million (ppm).

$$Mass\;Accuracy\left(ppm\right)=\left(\frac{Measured\frac{m}{z}-Theoretical\frac{m}{z}}{Theoretical\frac{m}{z}}\right)\times 10^{6}$$

Peaks with secondary mass spectrometry (MS2) data were identified using a self-constructed traditional Chinese medicine (TCM) MS2 database and corresponding fragmentation patterns. The obtained data are categorized using the Classfire database.

### 3.4. Network Pharmacology

#### 3.4.1. Target Collection of Disease

The targets related to stress response were screened using the Online Mendelian Inheritance in Man (OMIM), PharmGKB, TTD and GeneCards databases. The targets collected from these databases were integrated to obtain the final set of disease-related targets.

#### 3.4.2. Acquisition and Prediction of Active Ingredients and Corresponding Targets of *C. Rhizoma*

The active components of *C. Rhizoma* were sourced from the Traditional Chinese Medicine Systems Pharmacology Database TCMSP: https://tcmsp-e.com/ (accessed on 29 November 2024). The screening of these components was conducted using pharmacokinetic parameters, setting the standards for oral bioavailability (OB) at ≥30% and the drug-likeness (DL) value at ≥0.18. The OB and DL values are crucial screening criteria in drug discovery. OB reflects a drug’s ability to reach systemic circulation, with a higher OB indicating better absorption and efficacy. In TCMSP, compounds with OB ≥ 30% are more likely to achieve therapeutic concentrations. DL evaluates structural similarity to known drugs using the Tanimoto coefficient, with 0.18 as a threshold. A DL ≥ 0.18 suggests favorable pharmacokinetic properties, indicating a higher likelihood of successful development [73].

Following this, the Target Prediction function of the TCMSP platform and the Swiss Target Prediction platform http://www.swissadme.ch/ (accessed on 29 November 2024) were employed to predict the targets of active ingredients.

#### 3.4.3. C. Rhizoma Active Ingredient–Target–Disease Network and Protein Interaction Network

Venny 2.1.0 https://bioinfogp.cnb.csic.es/tools/venny/ (accessed on 29 November 2024) was used to map and identify overlapping targets between the active ingredient–target dataset and disease–target dataset. The STRING database was employed to study protein–protein interactions (PPIs). The *C. Rhizoma* active ingredient–target–disease network dataset was input into the Multiple Proteins TAB of STRING, with Homo sapiens selected as the species. Further analysis was conducted using Cytoscape 3.7.1 https://www.cytoscape.org/ (accessed on 29 November 2024), where topological parameters were calculated. The degree value of each node was used to set parameters in the PPI diagram, enhancing visualization. Core targets were identified based on degree centrality (DC), closeness centrality (CC), and betweenness centrality (BC).

#### 3.4.4. GO Enrichment Analysis and KEGG Pathway Analysis

To elucidate the molecular mechanisms underlying the anti-stress response of active ingredients in *C. Rhizoma*, Gene Ontology (GO) and Kyoto Encyclopedia of Genes and Genomes (KEGG) analysis were performed using the Metascape platform https://metascape.org/ (accessed on 29 November 2024). Core targets identified from the protein interaction network were inputted into Metascape, with Homo sapiens selected as the species. We conducted personalized analyses for the biological process (BP) and KEGG analysis. The results were downloaded and organized for further interpretation.

### 3.5. C. elegans Strains and Culture

The following *C. elegans* strains were obtained from the Caenorhabditis Genetic Center (University of Minnesota, Minneapolis, MN, USA): Bristol N2 (wild-type), TJ356 (zIs356[*daf-16p*::*daf-16a/b*::GFP + *rol-6*(*su1006*)]) GFP expression driven by the *daf-16* gene promoter. CF1553 (muIs84[(pAD76) *sod-3p*::GFP + *rol-6*(*su1006*)]), GFP driven by the *sod-3* promoter; *sod-3* is a target gene of *daf-16* and can be used to study oxidative stress resistance. CF1588 (*daf-16*(mu86) I; *daf-2*(e1370) III; muIs84 [(pAD76) *sod-3p*::GFP + rol-6(su1006)]); its *sod-3* gene is labeled with the GFP reporter gene and is a double knockout mutant of *daf-2* and *daf-16*. CF1038 (*daf-16*(mu86) I) is a *daf-16* mu86 deletion mutant, resulting in the loss of *daf-16* gene function.

The nematode growth medium (NGM) was prepared according to previously described methods [71]. *C. Rhizoma* stock solution was added to NGM to achieve final concentrations of 2.5 mg/mL, 5 mg/mL and 10 mg/mL. On the second day of preparation, When the OD600 of *Escherichia coli* (*E. coli*) OP50 reached 0.4–0.6, sterilized *C. Rhizoma* stock solution was added to the bacterial culture to achieve final concentrations of 2.5 mg/mL, 5 mg/mL, and 10 mg/mL.

### 3.6. Stress Resistance Analysis

Wild-type *C. elegans* at the oviposition stage were selected and treated synchronously. Two days later, N2 nematodes were subjected to the following stress treatments [74,75,76].

Ultraviolet (UV) Stress: The nematodes cultured in medium were exposed to UV irradiation at an intensity of 1000 J/cm^2^ for 8 min and 40 s. After irradiation, nematodes were transferred to fresh NGM and cultured at 20 °C. Survival was monitored and recorded every 12 h until all nematodes expired.

Heat Stress: After synchronization, nematodes were transferred to a 35 °C incubator and cultured for 48 h. Survival was monitored and recorded hourly until all nematodes died.

Oxidative Stress: After synchronization, nematodes from both the control group and drug-treated groups were transferred to NGM containing 240 μM juglone. Survival status was recorded hourly until all nematodes died.

Pathogen Stress: Nematodes were transferred to NGM agar plates containing *Pseudomonas aeruginosa* (*P. aeruginosa)* and their survival was recorded every 1 h until all nematodes expired.

### 3.7. Detection of Effects of C. Rhizoma on UV Resistance in Mutant Nematodes

Thirty CF1588 (*daf-16*(mu86) I; *daf-2*(e1370) III; muIs84) and CF1038 (*daf-16*(mu86) I) nematodes at the oviposition stage were synchronically treated on NGM for 2 h. After 72 h of incubation at 20 °C, 120 nematodes were transferred to either control or drug-treated medium. After an additional 48 h, the nematodes were subjected to UV stress treatment. Survival was monitored and recorded every 12 h until all nematodes expired [77].

### 3.8. Nucleus Localization Analysis of DAF-16

Thirty oviposition TJ356 nematodes (zIs356[*daf-16p*::*daf-16a/b*::GFP + *rol-6*(*su1006*)]) were synchronically treated on NGM for 2 h. After 72 h of incubation at 20 °C, 120 nematodes were transferred to either control or experimental medium. Following an additional 48 h of incubation, the nematodes were subjected to UV stress treatment and subsequently anesthetized with imidazole. Fluorescence was observed under an inverted fluorescence microscope, and DAF-16 nuclear translocation was measured and photographed [78].

### 3.9. Measurement of SOD-3 Expression in Nematodes

CF1553 nematodes genotypes (muIs84 [(pAD76) *sod-3p*::GFP + *rol-6*(*su1006*)]) have the *sod-3* gene tagged with the GFP reporter. CF1588 nematodes, a double-deletion mutant of *daf-2* and *daf-16*, also have the *sod-3* gene linked to GFP. Thirty CF1553 and CF1588 nematodes at the oviposition stage were synchronically treated on NGM for 2 h. After incubating at 20 °C for 72 h, 120 nematodes were transferred to either control or experimental medium. After an additional 48 h, the nematodes underwent UV stress treatment and were briefly anesthetized with levamisole hydrochloride. Ten nematodes from each group were selected for the fluorescence observation under an inverted fluorescence microscope [79].

### 3.10. Detection of ROS Content in Nematodes

Thirty wild-type nematodes at the spawning stage were selected and synchronously treated on NGM for 2 h. After 72 h, nematodes were transferred to the control group and experimental media for an additional 48 h. Then, nematodes from both groups were subjected to UV stress. They were subsequently washed three times with M9 buffer and stained with the DCFH-DA probe under dark conditions for 1 h. Ten nematodes were placed in each well of a black 96-well plate. A multifunctional enzyme marker was used for the measurement, with an excitation wavelength of 488 nm and an emission wavelength of 525 nm.

### 3.11. Assay of Superoxide Dismutase (SOD) Activity

Thirty wild-type C. elegans at the egg-laying stage were selected and synchronized on NGM plates for 3 h; after 72 h, 500 worms were transferred to both control and drug-treated media for cultivation, and following an additional 48 h, the worms from both groups were exposed to UV stress. They were then washed three times with M9 buffer and transferred to 1.5 mL EP tubes, discarding as much M9 buffer as possible in the final wash. Subsequently, 0.5 μL of 1 mM PMSF was added and the volume was adjusted to 500 μL with M9 buffer. The samples were subjected to three freeze–thaw cycles at −80 °C, each lasting 10 min, before being homogenized using an ultrasonic cell disruptor (400 W power, 6 s on/3 s off, total duration 20 min). Finally, the homogenate was centrifuged at 12,000 rpm for 15 min at 4 °C, and the supernatant was collected for the SOD enzyme activity assay using the Solarbio kit (Beijing, China) according to the manufacturer’s instructions.

### 3.12. Quantitative Real-Time PCR (qPCR)

L4-stage *C. elegans* (N2) were cultured on NGM agar plates with or without *C. Rhizoma* for two days. Then, the worms were exposed to UV radiation and transferred to new NGM agar plates. Total RNA was extracted using the Trizol method, followed by reverse transcription into cDNA using a reverse transcription kit (TransGen, Beijing, China), qPCR was performed with TransStart Top Green qPCR Super Mix (TransGen, Beijing, China) on a PCR instrument, using act-1 (actin) as the reference gene. Gene expression differences were analyzed using the ∆∆CT method. Primers were designed using Primer 5 (see Table 3).

### 3.13. Effects of C. Rhizoma on Health Indexes of Nematodes

Thirty wild-type nematodes at the spawning stage were synchronized for 2 h in either control or drug group media. Day 0 was designated as the end of synchronization. Starting from day 3, nematode survival was monitored daily, with plates rotated every 24 h until all nematodes perished. Nematode movement states were categorized as follows: Type A, autonomous movement without stimulation; Type B, movement upon external stimulation; Type C, limited movement (head or tail) upon stimulation; and Type D, deceased nematodes. Additionally, starting from day 3, the pharyngeal pumping rate of nematodes was observed at fixed times daily by counting the number of pumps within 1 min. Another set of thirty wild-type nematodes at the spawning stage were synchronized for 2 h in either the control group or drug group media. After synchronization, nematodes were transferred individually to fresh media, with 10 parallel groups per treatment. This was repeated daily until no more eggs were laid. After 24 h of growth, media were refrigerated at 4 °C for 1 h, followed by the microscopic counting of nematodes.

### 3.14. Molecular Docking

To further identify the mechanisms of key components in the water extract of *C. Rhizoma*, molecular docking was conducted to screen for effective compounds. The structures of dihydropyrocurzerenone, pyrocurzerenone, tetrahydrocurcumin, hexahydrocurcumin, octahydrocurcumin, curcumenol, procurcumenol, isoprocurcumenol, curcumadione and germacrone were retrieved from the PubChem Database http://pubchem.ncbi.nlm.nih.gov (accessed on 29 November 2024). The crystal structure of FOXO (PDB ID: 6QZS) was obtained from the RCSB Protein Data Bank http://www.rcsb.org (accessed on 29 November 2024). Ligand and protein preparations, as well as the molecular docking process, were carried out using Discovery Studio 2019 software.

## 4. Conclusions

In summary, *C. Rhizoma* aqueous extract significantly enhanced the stress resistance of nematodes, including UV, heat, oxidative, and *Pseudomonas aeruginosa*-induced stress. It reduced ROS levels after UV exposure, mitigating oxidative damage, and increased SOD expression and activity. The involvement of the insulin signaling pathway, particularly the *daf-2* and *daf-16* genes, was confirmed through studies with mutant nematodes. Additionally, the nuclear translocation of DAF-16 provided an insight into the underlying mechanism of action. Network pharmacology predictions, UPLC-MS/MS analysis, and molecular docking to FoxO revealed curcuminoids and dihydropyrocurzerenone as key active compounds likely responsible for these effects of the *C. Rhizoma* aqueous extract. Furthermore, *C. Rhizoma* extended the lifespan of nematode, improved movement and swallowing abilities in elderly nematodes, and positively impacted healthspan. Overall, *C. Rhizoma,* as a traditional Chinese medicinal herb, exhibited remarkable anti-stress and anti-aging properties, largely attributable to its multiple bioactive compounds. Future studies will further explore the specific molecular mechanisms of these compounds to elucidate the distinct contributions of each component to the overall therapeutic effect, thereby providing a more robust theoretical foundation for its clinical application.

## Figures and Tables

**Table 1:** Active ingredients of *C. Rhizoma* water extract.

CAS./No.	Ingredient of *C. Rhizoma* *	Formula	Ingredient of *C. Rhizoma* Water Extract ^#^	Formula	Retention Time	*m*/*z* Found	Mass Error (ppm)
19431-84-6	Curcumenol	C_15_H_22_O_2_	Curcumenol	C15H22O2	10.41	235.1691	0.5
			Procurcumenol	C15H22O2	11.50	217.1587	0.2
			Isoprocurcumenol	C15H22O2	9.23	217.1587	0.1
			Curcumadione	C15H22O2	10.25	235.1690	0.7
458-37-7	Curcumin	C_21_H_20_O_6_	Tetrahydrocurcumin	C21H24O6	8.60	355.1541	0.4
			Hexahydrocurcumin	C21H26O6	6.39	357.1697	0.3
			Octahydrocurcumin	C21H28O6	7.63	341.1745	−0.4
20085-85-2	Epicurzerenone	C_15_H_18_O_2_	Dihydropyrocurzerenone Pyrocurzerenone	C15H18O	10.65	215.1431	0.3
				C15H16O	8.35	213.1274	0.3
20303-60-0	Germacrone	C_15_H_22_O	Germacrone	C_15_H_22_O	9.68	219.1744	0.6

* The active ingredients of *C. Rhizoma* predicted from the TCMSP and Swiss ADME platforms. ^#^ The components in the aqueous extract of *C. Rhizoma* detected using UPLC-MS/MS.

**Table 2:** Active ingredient–target–disease dataset core target.

Target ID	Degree	Betweenness Centrality	Closeness Centrality
AKT1	91	0.09649359	0.77300613
TNF	85	0.07968469	0.74117647
EGFR	74	0.04810785	0.7
SRC	74	0.06556073	0.70391061
HSP90AA1	73	0.03490242	0.68108108
MAPK3	72	0.03348627	0.68852459
STAT3	71	0.0303438	0.67379679
MTOR	66	0.0192511	0.65284974
ESR1	61	0.03135785	0.64615385
CCND1	57	0.01591554	0.62376238
PTGS2	56	0.01927096	0.62686567
EP300	54	0.02552771	0.61764706
PPARG	52	0.02037604	0.61463415
MMP9	52	0.01095982	0.60287081
RELA	50	0.01085425	0.60576923
PIK3CA	49	0.00798017	0.59433962
TLR4	46	0.01037593	0.59433962
MDM2	43	0.00577946	0.58333333
MAPK14	42	0.0046569	0.58064516
PPARA	41	0.0158975	0.58333333
GSK3B	41	0.00556238	0.57798165
JAK2	40	0.00410697	0.57013575
AR	37	0.00515632	0.56756757
MAP2K1	37	0.00294822	0.56
CDK4	36	0.00395323	0.55752212
PRKCA	36	0.00522523	0.56502242
APP	35	0.01207911	0.56756757
LYN	35	0.00356585	0.54077253
RAF1	35	0.00410561	0.55021834
NR3C1	34	0.019599	0.57013575
PTPN11	34	0.00315151	0.54782609
ABL1	33	0.00363776	0.55021834
CDK2	33	0.00300429	0.54545455
LCK	33	0.00351632	0.55263158
HMOX1	33	0.00369027	0.55263158
PLCG1	32	0.00376055	0.54310345
PARP1	32	0.00320413	0.54310345
JAK1	30	0.00363108	0.525
ACE	30	0.00980281	0.55506608
PGR	30	0.0046664	0.55506608
PRKCB	29	0.00545351	0.53389831

**Table 3:** Primer sequence.

Gene	Forward Primer	Reverse Primer
*act-1*	5′-GTCATGGTCGGTATGGGACA-3′	5′-TTCGTAGATTGGGACGGTGT-3′
*daf-16*	5′-TTTCCGTCCCCGAACTCAA-3′	5′-ATTCGCCAACCCATGATGG-3′
*hsf-1*	5′-TTGACGACGACAAGCTTCCAGT-3′	5′-AAAGCTTGCACCAGAATCATCCC-3′
*hsp-16.1*	5′-CCACTATTTCCGTCCAGCTC-3′	5′-TGGAGAGCCTCTGCAAACTG-3′
*sod-3*	5′-CTCTTTTGGGAGGAAGTTATGG-3′	5′-GCCAGTTGGTCAGAAGATAG-3′
*hsp-16.2*	5′-CTGCAGAATCTCTCCATCTGAGTC-3′	5′-AGATTCGAAGCAACTGCACC-3′
*hsp-16.49*	5′-GTCAAATCTGCAATTTCGAATG-3′	5′-CAAATAATGGGATAGAAGAG-3′
*hsp-12.6*	5′-TGGCCACTTCAAAAGGGAG-3′	5′-CTCTTTTGGAGGAAGTATGG-3′

## Data Availability

The original contributions presented in this study are included in the article and Supplementary Material, and further inquiries are available from the corresponding authors.

## Supplementary Materials

The following supporting information can be downloaded at: https://www.mdpi.com/article/10.3390/molecules30081668/s1, Figure S1: Classification of components in the aqueous extract of *C. Rhizoma* based on Classfire database comparison; Figure S2: The aqueous extract of *C. Rhizoma* contains 766 metabolites, of which 282 were detected in negative ion mode. Figure S3: 484 metabolites in the aqueous extract of *C. Rhizoma* were detected in positive ion mode, while curcumadione dominates in the positive ion mode. Figure S4: Intersection genes between active components of *C. Rhizoma* and disease targets. Figure S5: Top-ranked core nodes in the protein-protein interaction network of intersection targets within the *C. Rhizoma* active ingredient-target-disease dataset. Figure S6: The statistical graph displays quantitative counting of DAF-16 nuclear translocation. Figure S7: Molecular docking of (A) Pyrocurzerenone, (B) Germacrone, (C) Curcumadione, (D) Isoprocurcumenol, (E) Procurcumenol, and (F) Curcumonel with the target protein FoxO, accompanied by their respective 2D schematic representations. Table S1: Active ingredients from Swiss ADME; Table S2: Active ingredients from TCMSP.

## Abbreviations

TCM, traditional Chinese medicine; *C. elegans*, *Caenorhabditis elegans*; GO, Gene Ontology; KEGG, Kyoto Encyclopedia of Genes and Genomes; ROS, reactive oxygen species; SOD, superoxide dismutase; NGM, nematode growth medium; THC, tetrahydrocurcumin; HHC, hexahydrocurcumin; and OHC, octahydrocurcumin.
