# Supplementary material for: Unlocking the Potential of Curcumae Rhizoma Aqueous Extract in Stress Resistance and Extending Lifespan in Caenorhabditis elegans
Source: Molecules. 2025 Apr 8;30(8):1668. doi: 10.3390/molecules30081668 (PMC12029441; doi:10.3390/molecules30081668)
Supplement: Supplementary file 1 [file molecules-30-01668-s001.zip › molecules-3496618-supplementary.pdf]

Unlocking the Potential of *Curcumae Rhizoma* Aqueous Extract in Stress Resistance and Extending Lifespan in *Caenorhabditis elegan*

Supplemental Materials

Table S1. Active ingredients from Swiss ADME

CAS.	Ingredient	Pharmacokinetics	Druglikeness
20085-85-2	epicurzerenone	YES	YES
489-79-2	linderazulene	YES	YES
22608-11-3	demethoxycurcumin	YES	YES
458-37-7	curcumin	YES	YES
83217-89-4	1β-Hydroxy-β-eudesmol	YES	YES
13657-68-6	curdione	YES	YES
57566-47-9	furanodiene	YES	YES
19431-84-6	curcumenol	YES	YES
20303-60-0	germacrone	YES	YES
18486-69-6	myrtenal	YES	YES
85710-39-0	cedrenol	YES	YES
481-34-5	α-cadinol	YES	YES
481-18-5	α-spinasterol	YES	YES
100347-96-4	curcumenone	YES	YES
25679-28-1	(Z)-anethole	YES	YES

Table S2. Active ingredients from TCMSP

NO.	Ingredient	OB/%	DL
MOL000296	hederagenin	36.91	0.75
MOL000906	wenjine	47.93	0.27
MOL000940	bisdemethoxycurcumin	77.38	0.26

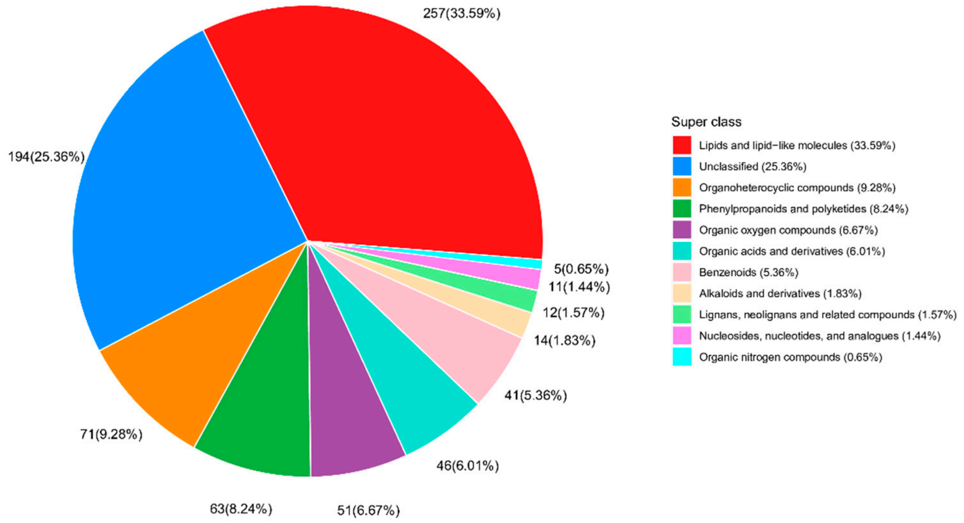

Figure S1. Classification of components in the aqueous extract of *C. Rhizoma* based on Classfire database comparison: 33.59% lipids, 9.28% organic heterocyclic compounds, and 8.24% phenylpropanoids and polyketides.

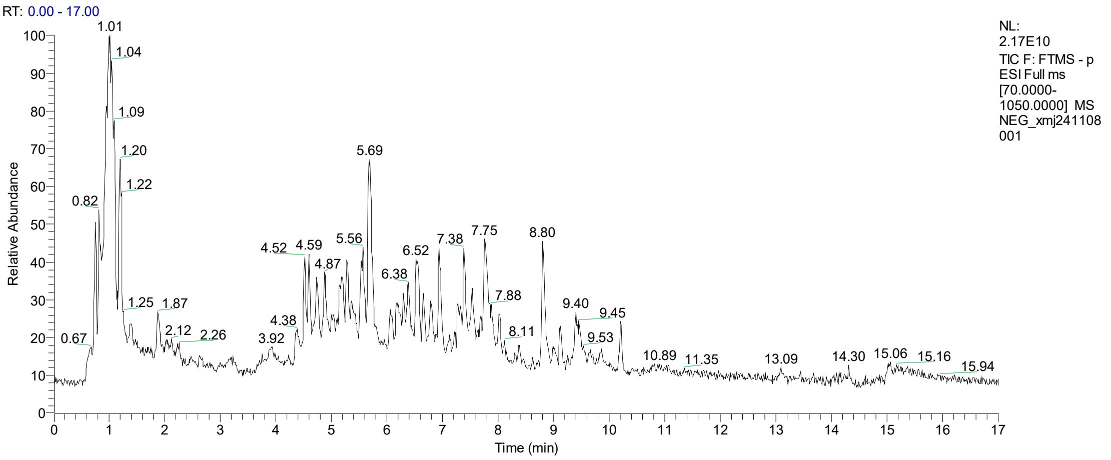

Figure S2. The aqueous extract of *C. Rhizoma* contains 766 metabolites, of which 282 were detected in negative ion mode. Among these, citric acid exhibits the highest relative content in the negative ion mode.

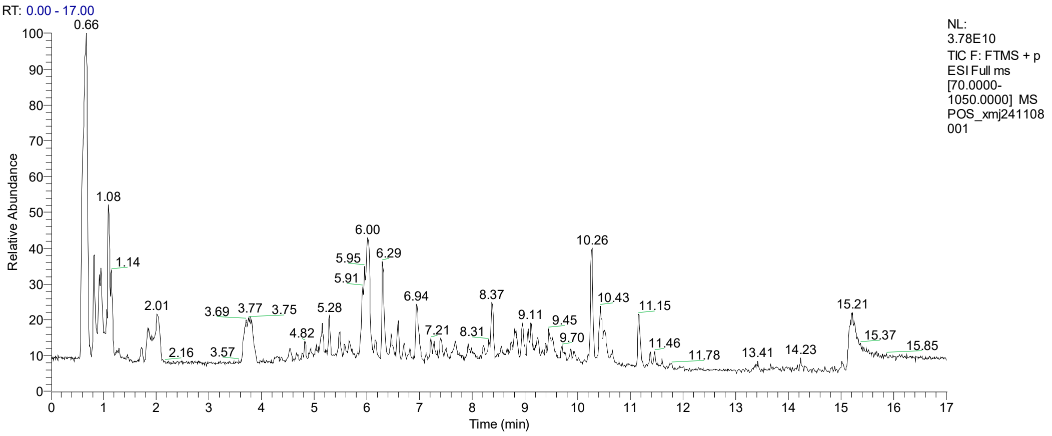

Figure S3. 484 metabolites in the aqueous extract of *C. Rhizoma* were detected in positive ion mode, while curcumadione dominates in the positive ion mode.

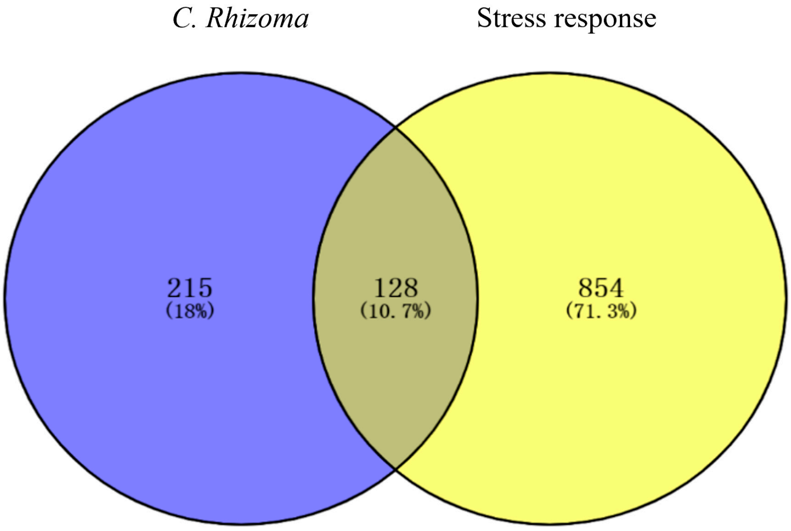

Figure S4. Intersection genes between active components of *C. Rhizoma* and disease targets.

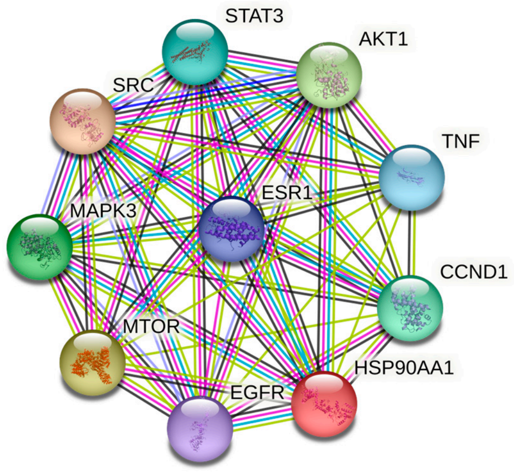

Figure S5. Top-ranked core nodes in the protein-protein interaction network of intersection targets within the *C. Rhizoma* active ingredient-target-disease dataset.

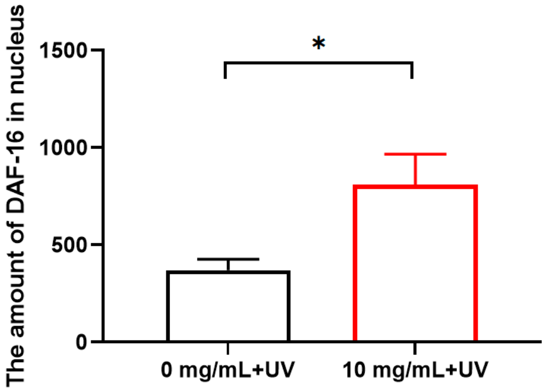

Figure S6. The statistical graph displays quantitative counting of DAF-16 nuclear translocation.

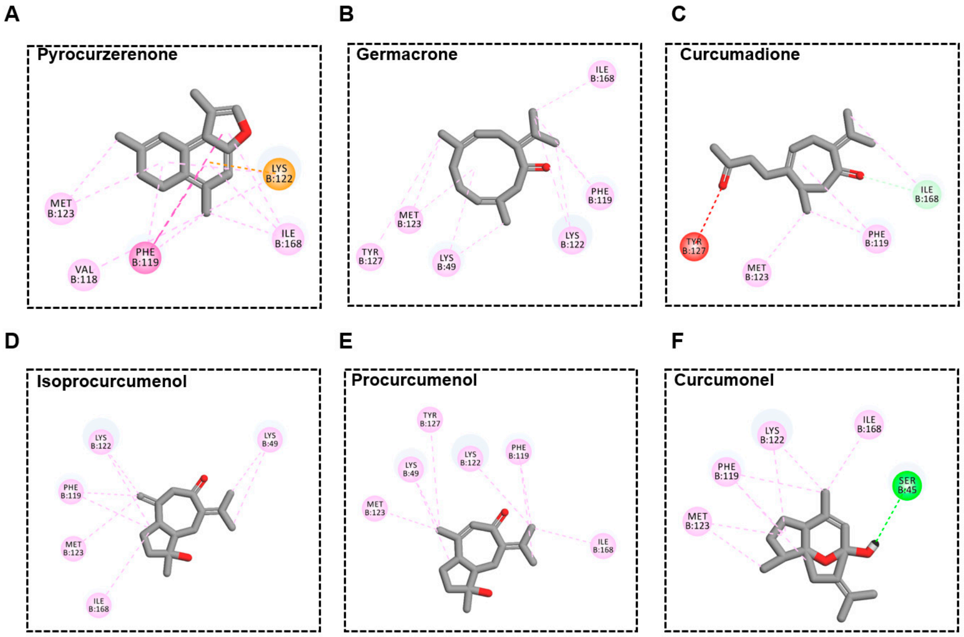

Figure S7. Molecular docking of (A) Pyrocurzerenone, (B) Germacrone, (C) Curcumadione, (D) Isoprocurcumenol, (E) Procurcumenol, and (F) Curcumonel with the target protein FoxO, accompanied by their respective 2D schematic representations.
